# Supplementary material for: Content-rich biological network constructed by mining PubMed abstracts
Source: BMC Bioinformatics. 2004 Oct 8;5:147. doi: 10.1186/1471-2105-5-147 (PMC528731; doi:10.1186/1471-2105-5-147)
Supplement: Additional File 2 — The original results of the above study (non-essential files are deleted to keep the file size under the limit set by BMC bioinformatics). [file 1471-2105-5-147-S2.bz2 › chilibotAdditionalFile2/dip30/745ID7744737E18125/html/TOLA_TOLR.html]

 


 **TOLA** and **TOLR** 
  
Found 33 abstracts in PubMed, retrieved 30.  
 

 What does Google say? 
 PDF only 
| .edu only 

---

**Interactive relationship** (e.g. stimulation, inhibition, etc)

**Neutral relationship**- The TolQ  **TolR**  proteins energize  **TolA**  and share homologies with the flagellar motor proteins MotA MotB.  Ref: 11722743 Mol Microbiol, 2001
- These data demonstrate that  **TolA**  interacts directly with  **TolR**  and TolQ.  Ref: 7744737 J Biol Chem, 1995
- The role of the  **TolR**  C terminal domain in the  **TolA**   **TolR**  interaction and its association with the membranes was also demonstrated.  Ref: 10419942 J Bacteriol, 1999
- These results confirmed the essential role of the transmembrane domain of  **TolA**  in triggering interactions with TolQ and  **TolR** .  Ref: 9851983 J Bacteriol, 1998
- Cross linking experiments have shown that the  **TolA**  transmembrane domain interacts with TolQ and  **TolR** .  Ref: 10419942 J Bacteriol, 1999
- **TolA**  N terminal domain interacts with TolQ and  **TolR**  proteins.  Ref: 7744737 J Biol Chem, 1995
- Moreover, immunoprecipitation experiments with the antiserum directed against  **TolA**  indicated that TolQ and  **TolR**  were co immunoprecipitated with  **TolA**  after cross linking.  Ref: 7744737 J Biol Chem, 1995
- Role of  **TolR**  N terminal, central, and C terminal domains in dimerization and interaction with  **TolA**  and tolQ.  Ref: 10419942 J Bacteriol, 1999
- The N terminal transmembrane domain of  **TolA**  anchors the protein to the cytoplasmic membrane and interacts with TolQ and  **TolR** .  Ref: 9851983 J Bacteriol, 1998
- Energy dependent conformational change in the  **TolA**  protein of Escherichia coli involves its N terminal domain, TolQ, and  **TolR** .  Ref: 11418549 J Bacteriol, 2001
- In addition, analyses of the beta galactosidase activities of different translational fusion constructs revealed that translation of  **tolA**  and orf2 genes was dependent on the translation of their corresponding upstream genes  **tolR**  and oprL, respectively.  Ref: 12486055 J Bacteriol, 2003

**Non-interactive relationship** (e.g. studied together, co-existance, homology, etc.)

- Mutations affecting the function of  **TolA**  resulted in a lack or an alteration in  **TolA**  TolQ or  **TolR**   **TolA**  interactions but did not affect the formation of TolQ  **TolR**  complexes.  Ref: 9851983 J Bacteriol, 1998
- The  **TolA** , TolB, TolQ, and  **TolR**  proteins are involved in maintaining the integrity of the Escherichia coli outer membrane and in the import of group A colicins and filamentous phage DNA.  Ref: 7744737 J Biol Chem, 1995
- However, Pal was present in larger amounts compared to  **TolA**  and  **TolR**  proteins.  Ref: 11790745 J Bacteriol, 2002
- The nucleotide sequence of a 2.9 kb DNA fragment containing the tolQ,  **tolR** , and  **tolA**  genes was determined.  Ref: 8955385 J Bacteriol, 1996
- TolQ,  **TolR** , and  **TolA**  are located in the cytoplasmic membrane where they interact with each other via their transmembrane domains.  Ref: 10633120 J Bacteriol, 2000
- However, strains with mutations in both tcpA and either tolQ,  **tolR** , or  **tolA**  were completely resistant to CTXphi infection.  Ref: 10692381 J Bacteriol, 2000
- This cluster was found to consist of seven genes in the order orf1 tolQ  **tolR**   **tolA**  tolB oprL orf2.  Ref: 10735848 J Bacteriol, 2000
- Suppressor mutant analyses have localized the TolQ  **TolA**  interaction to the first transmembrane domain of TolQ and have shown that the third transmembrane domain of TolQ interacts with the transmembrane domain of  **TolR** .  Ref: 10419942 J Bacteriol, 1999
- Mutations in tolQ,  **tolR** ,  **tolA** , tolB, or pal genes result in sensitivity to bile salts and the leakage of periplasmic proteins.  Ref: 10633120 J Bacteriol, 2000
- In this study, we show that both TolQ and  **TolR**  proteins are essential for the  **TolA**  Pal interaction.  Ref: 11722743 Mol Microbiol, 2001
- In the present study we cloned and mutagenized the orf1, tolQ,  **tolR** ,  **tolA** , and tolB genes from P. putida KT2440, which were located upstream of the oprL gene.  Ref: 10940016 J Bacteriol, 2000
- Activity of ExbB ExbD via  **TolA**  was higher in cells lacking TonB, and activity of TolQ  **TolR**  via TonB was increased when  **TolA**  was missing.  Ref: 8316079 Mol Microbiol, 1993
- The tolQ,  **tolR** , and  **tolA**  genes from Pseudomonas aeruginosa PAO were cloned using degenerate oligonucleotide PCR primers designed based on conserved transmembrane regions of Escherichia coli TolQ and  **TolR**  and E. coli and Pseudomonas putida ExbB and ExbD.  Ref: 8955385 J Bacteriol, 1996
- Modifying the Asp residue within the TM region of  **TolR**  indicated that an acidic residue was important for the pmf dependent interaction of  **TolA**  with Pal and outer membrane stabilization.  Ref: 11722743 Mol Microbiol, 2001
- This infection is dependent on an intact particle and requires the products of the tolQ,  **tolR** , and  **tolA**  genes of the bacteria.  Ref: 3053655 J Bacteriol, 1988
- Group B colicins, ferric siderophores and vitamin B12 are taken up via the TonB ExbB ExbD, group A colicins via the  **TolA**  TolQ  **TolR**  system.  Ref: 8316079 Mol Microbiol, 1993
- The deduced amino acid aa sequence of the H. influenzae TolQ,  **TolR** ,  **TolA**  and TolB show 6 1 and 62% identity with Escherichia coli TolQRAB proteins, respectively.  Ref: 8921895 Gene, 1996
- Transmembrane domains of TolQ,  **TolR**  and  **TolA**  interact in the cytoplasmic membrane, while TolB and Pal form a complex near the outer membrane.  Ref: 9701827 Mol Microbiol, 1998
- The  **TolR**  transmembrane domain was shown to be involved in the  **TolA**   **TolR**  and TolQ  **TolR**  interactions, while  **TolR**  central and C terminal domains appeared to be involved in  **TolR**  dimerization.  Ref: 10419942 J Bacteriol, 1999
- It is organized into two complexes, one near the outer membrane between TolB and PAL and one in the cytoplasmic membrane between  **TolA** , TolQ, and  **TolR** .  Ref: 10419942 J Bacteriol, 1999
- In this study, we demonstrate that the genes tolQ,  **tolR** , and  **tolA** , but not tolB, tolC, and ompF, are required for the internalization of cloacin DF13 and they are not involved in the transport of ferric aerobactin.  Ref: 8419302 J Bacteriol, 1993
- **TolA** , TolQ, and  **TolR**  are inner membrane proteins, TolB is a periplasmic protein, and Pal, the peptidoglycan associated lipoprotein, is anchored to the outer membrane.  Ref: 11790745 J Bacteriol, 2002
- **TolA** , TolQ, and  **TolR**  are localized in the inner membrane while TolB is periplasmic, although a small amount of membrane associated TolB is always found.  Ref: 7744737 J Biol Chem, 1995
- Evolutionary relationship of uptake systems for biopolymers in Escherichia coli cross complementation between the TonB ExbB ExbD and the  **TolA**  TolQ  **TolR**  proteins.  Ref: 8316079 Mol Microbiol, 1993
- This change requires TolQ,  **TolR** , and a functional  **TolA**  N terminal domain.  Ref: 11418549 J Bacteriol, 2001
- Group A colicins need proteins of the Escherichia coli envelope Tol complex  **TolA** , TolB, TolQ and  **TolR**  to reach their cellular target.  Ref: 9466263 Mol Microbiol, 1998
- Analysis of polar mutations, reverse transcriptase PCR assays, and transcriptional fusion constructs with a promoterless lacZ gene revealed that the genes are arranged in two operons orf1 tolQ  **tolR**   **tolA**  tolB and oprL orf2.  Ref: 12486055 J Bacteriol, 2003
- Bacteria carrying  **tolA** , tolQ and  **tolR**  mutations kept their colicin insensitivity as spheroplasts, just as the resistant ones.  Ref: 12094727 Folia Microbiol Praha , 2002
- Since these two  **TolA**  derivatives still formed specific complexes with  **TolR**  derivatives still formed specific complexes with  **TolR**  and TolQ, we concluded that the  **TolA**  N terminal domain interacted with these proteins.  Ref: 7744737 J Biol Chem, 1995
- The Tol Pal system of the Escherichia coli envelope is formed from the inner membrane TolQ,  **TolR**  and  **TolA**  proteins, the periplasmic TolB protein and the outer membrane Pal lipoprotein.  Ref: 11115123 Mol Microbiol, 2000
- The interactions of  **TolR**  with  **TolA**  and TolQ were confirmed,  **TolR**  was shown to dimerize, and the resulting dimer was shown to interact with TolQ.  Ref: 10419942 J Bacteriol, 1999
- It was shown that the  **TolA** , TolQ, and  **TolR**  proteins play a critical role in the translocation of group A colicins and filamentous bacteriophage minor coat proteins g3p.  Ref: 11851406 Biochemistry, 2002
- Polar and nonpolar mutations of the P. putida tolQ,  **tolR** ,  **tolA** , and tolB genes were generated in vitro by using the omega Km r interposon, which carries two transcriptional stop signals, or a promoterless xylE cassette, lacking any transcriptional stop signal, respectively.  Ref: 10940016 J Bacteriol, 2000
- The TolQ,  **TolR** ,  **TolA** , TolB, and Pal proteins appear to function in maintaining the integrity of the outer membrane, as well as facilitating the uptake of the group A colicins and the DNA of the infecting filamentous bacteriophages.  Ref: 8763928 J Bacteriol, 1996
- The Tol Pal system of Escherichia coli is composed of the YbgC, TolQ,  **TolA** ,  **TolR** , TolB, Pal and YbgF proteins.  Ref: 11994151 Mol Microbiol, 2002
- In Pseudomonas putida, the seven genes, orf1, tolQ,  **tolR** ,  **tolA** , tolB, oprL, and orf2, which encode the proteins of this complex, are clustered in a 5.8 kb region of chromosomal DNA.  Ref: 12486055 J Bacteriol, 2003
- The TolQ  **TolR**   **TolA**  proteins form a complex in the cytoplasmic membrane, while TolB and Pal interact near the outer membrane.  Ref: 10474183 FEMS Microbiol Lett, 1999
- **TolA**  and  **TolR**  are anchored to the inner membrane by a single transmembrane domain, TolQ is an integral membrane protein with three transmembrane segments, and TolB has recently been found to be periplasmic although it is partially membrane associated.  Ref: 7744736 J Biol Chem, 1995
- TolQ,  **TolR**  and  **TolA**  are membrane proteins involved in maintaining the structure of Escherichia coli cell envelope.  Ref: 7853390 J Mol Biol, 1995
- Transmembrane domains of TolQ,  **TolR** , and  **TolA**  interact in the cytoplasmic membrane, while TolB and Pal form a complex near the outer membrane.  Ref: 9851983 J Bacteriol, 1998
- Most of the amino acid residues of  **TolA** , TolB,  **TolR**  and Pal are localized in the periplasm.  Ref: 10474183 FEMS Microbiol Lett, 1999
- The resulting PCR product was used as a probe to isolate a 6.5 kb DNA fragment containing P. aeruginosa tolQ,  **tolR** , and  **tolA** .  Ref: 8955385 J Bacteriol, 1996
- Isolation and characterization of the Haemophilus influenzae tolQ,  **tolR** ,  **tolA**  and tolB genes.  Ref: 8921895 Gene, 1996
- Subsequent translocation of phage DNA requires the chromosomally encoded TolQ,  **TolR** , and  **TolA**  proteins, after the pilus presumably has withdrawn, bringing the phage to the bacterial surface.  Ref: 9335297 J Bacteriol, 1997
- V. cholerae strains with insertion mutations in tolQ,  **tolR** , or  **tolA**  were reduced in their efficiency of CTXphi uptake by 4 orders of magnitude.  Ref: 10692381 J Bacteriol, 2000
- Sequence data showed that these genes are clustered in a 6 kb segment of DNA with the gene order orf1 tolQ  **tolR**   **tolA**  tolB pal orf2 a newly identified open reading frame encoding a 29 kD9 protein.  Ref: 8763928 J Bacteriol, 1996
- TolQ,  **TolR** , and  **TolA**  inner membrane proteins of Escherichia coli are involved in maintaining the stability of the outer membrane.  Ref: 11418549 J Bacteriol, 2001
- DNA transfer requires the chromosomally encoded  **TolA** , TolQ, and  **TolR**  cytoplasmic membrane proteins.  Ref: 9537368 J Bacteriol, 1998
- TolQ  **TolR**  interacted through TonB, and ExbB ExbD interacted through  **TolA**  with the outer membrane receptors and colicins.  Ref: 8316079 Mol Microbiol, 1993
- The predicted amino acid sequences of V. cholerae TolQ,  **TolR** ,  **TolA** , and TolB showed significant similarity to the corresponding E. coli sequences.  Ref: 10692381 J Bacteriol, 2000
- This fragment was also detected in Delta tolQ, Delta  **tolR** , and  **tolA**  H22P mutants but, in contrast to the parental strain, was also seen in the absence of uncouplers.  Ref: 11418549 J Bacteriol, 2001
